# Integrated Experimental and Computational Investigation of Azobenzenoid Derivatives Against Trypanosomatid: RNA Editing Ligase 1 as a Potential Molecular Target

**DOI:** 10.1007/s11686-026-01391-1

**Published:** 2026-09-07

**Authors:** Asif Khan, Rodolfo Bento Balbinot, Danielle Lazarin-Bidóia, Maira Gabriela Paetzold, Íris Nunes Seixas, Gustavo Nunes Ramos Silva, Maria Aparecida Fernandez, Celso Vataru Nakamura, Flavio Augusto Vicente Seixas

**Affiliations:** 1 Laboratory of Structural Biochemistry, Department of Technology, State University of Maringá, Av. Ângelo Moreira da Fonseca, 1800, Pq Danielle, Umuarama, PR 87506-370 Brazil; 2 Technological Innovation Laboratory in the Pharmaceuticals and Cosmetics Development, State University of Maringá, Maringá, PR Brazil; 3 Department of Odontology, Paranaense University, Umuarama, PR Brazil; 4 Department of Medicine, University of São Paulo, São Paulo, SP Brazil; 5 Department of Biotechnology, Genetics and Cell Biology, State University of Maringá, Maringá, PR Brazil

**Keywords:** *Trypanosoma*, *Leishmania*, RNA editing ligase 1, Drug discovery, Molecular docking, MD simulation, Cytotoxicity

## Abstract

**Background:**

Neglected tropical diseases caused by pathogenic protozoa, including Chagas Disease (etiological agent Trypanosoma cruzi) and leishmaniasis (etiological agent Leishmania spp.), are public health problems in many developing countries.

**Aims and Methodolgy:**

Due to the lack of safe and effective therapies, the current study was designed to screen synthetic azobenzenoid derivatives using integrated in silico and in vitro assays.

**Results:**

Among the tested compounds, CaCS2 potentially inhibited promastigotes and amastigotes of *Leishmania amazonensis* and epimastigotes and trypomastigotes of *Trypanosoma* cruzi, while showing no cytotoxicity towards human J774A.1 and LLC-MK_2_ cell lines. Reverse docking identified the parasite-specific RNA-editing ligase 1 (REL1), completely absent in humans, as a high-confidence target (p-value = 1.76 × 10^-12^; MaxTC = 0.31). Similarly, docking simulations revealed high binding probabilities for CaCS2 in two main cavities of REL1, named the orthosteric and alternative sites, in both parasites. Molecular dynamic simulations confirmed stable CaCS2 binding at the orthosteric site, while interactions at the alternative site were transient.

**Conclusion:**

Interestingly, CaCS2 was a specific ligand to REL1 and non cytotoxic to human cell lines, making it a promising scaffold for anti-trypanosomatid development.

**Supplementary Information:**

The online version contains supplementary material available at https://doi.org/10.1007/s11686-026-01391-1.

## Introduction

Trypanosomatidae members i.e., *Trypanosoma* and *Leishmania* sp. are intracellular parasites that cause several complications, including sleeping sickness, Chagas Disease (CD), and leishmaniasis in both animals and humans [1]. Both parasites are vector-borne; transmission remains the major route of infection; however, congenital, transfusional, transplant-associated, and oral transmission are also commonly found in South America, Africa, the Middle East, and Asia, especially in developing countries, such as Brazil [2]. According to the Drugs for Neglected Diseases initiative (DNDi-2019), approximately 1.2 million cases are reported annually in over 90 countries, and Brazil is among the top six nations [3, 4]. Similarly, *T. cruzi* causes CD, which is prevalent in 21 nations in the Americas but can spread to other regions of the world through the migration of infected humans and animals [5]. According to the Pan American Health Organization (PAHO), globally, there are an estimated 6–7 million people infected with CD, among them, 30,000 new cases and 14,000 annual deaths [6]. It has been taken into consideration that around 95% of infected individuals die if not treated simultaneously. Furthermore, 70 million people are still at risk of getting CD [7–9].

Chagas disease (CD), also known as American trypanosomiasis, is caused by *Trypanosoma cruzi*, a highly diverse parasitic group/taxon, transmitted by arthropod vectors of the family Reduviidae, such as *Triatoma* insects [10, 11]. At the biting site, *Triatoma* excretes feces that contain *T. cruzi* protozoa, which are rubbed or crushed into the bite wound to reduce irritation [12]. After entering the bloodstream, the parasite damages internal organs, most commonly the intestines and heart. A wide range of neurological symptoms is observed in human American trypanosomiasis (HAT). This encompasses sleep, sensory, motor, and psychological abnormalities in addition to the distinctive sleep disturbances that have earned this illness the colloquial moniker “sleeping sickness” [13, 14].

Leishmaniasis is a heterogeneous parasitic disease caused by several *Leishmania* [9, 15] and manifests in four major clinical forms: localized cutaneous, followed by diffuse cutaneous, mucocutaneous, and visceral leishmaniasis (the most severe form) [16]. Among others, cutaneous leishmaniasis is primarily characterized by localized skin lesions or ulcers, whereas mucocutaneous disease can cause progressive destruction of mucosal tissues [17]. Generally, diffuse cutaneous leishmaniasis is associated with several health-related complications like skin lesions, while visceral leishmaniasis represents the most severe form and commonly presents with prolonged fever, hepatosplenomegaly, weight loss, and other related haematological abnormalities [18–20]. However, different symptoms appear in patients depending on the condition of the disease; for example, in the case of mucocutaneous lesions, upper respiratory congestion along with hoarseness, edema, epistaxis with erythematous and boggy mucosa with purulent drainage [21]. The available treatments vary according to the infecting species, clinical form, geographic region, and disease severity and may include pentavalent antimonial, amphotericin B formulation, meglumine, antimoniate, and sodium stibogluconate [22].

In contrast, Chagas disease, caused by the protozoan parasite *Trypanosoma cruzi*, presents with a heterogeneous clinical spectrum that can be broadly divided into two main states: acute and chronic phases [23, 24]. The acute phase is frequently asymptomatic conditions like, fever, fatigue, headache, lymphadenopathy, and localized inflammatory lesions at the site of the parasite bite or entry site [25]. Following the acute phase, most infected individuals enter an intermediate chronic phase, characterized by persistent parasitemia in the absence of evident cardiac or gastrointestinal manifestations [26]. Furthermore, chronic Chagas cardiomyopathy may manifest as conduction abnormalities, arrhythmias, ventricular dysfunction, heart failure, and thromboembolic events, whereas gastrointestinal complications may result in oesophageal or colonic dilation leading to dysphagia, regurgitation, constipation, and abdominal discomfort [27, 28]. The treatment of Chagas disease varies, depending on patients’ age, disease stage/condition, clinical manifestation, and geographic setting; however, in general, benznidazole and nifurtimox are usually used [29].

However, due to their poor therapeutic index, rise in drug resistance, and expensive medications, the majority of therapies for these conditions have significant toxicity and adverse effects [30]. To identify a potential drug candidate, various antimicrobial tests are conducted in laboratories under controlled conditions. Furthermore, the use of advanced bioinformatics tools and software for drug discovery is an interesting approach (fast, easy, reliable, and cost-effective), which further validates laboratory findings [31, 32].

Recent studies have demonstrated the efficacy of an azobenzenoid derivative compound named CaCS2 (PubChem id: 79465) as a potential inhibitor of the chorismate synthase (CS) enzyme [33]. CS is present in mycobacteria, fungi, plants, and apicomplexan parasites, making it an attractive and promising target for the development of novel antimicrobial agents [34–36]. Although CS is absent from trypanosomatid genomes, the current in vitro study identified that CaCS2 also exhibited potential activity against strains of *Trypanosoma cruzi* and *Leishmania amazonensis*. This finding is particularly intriguing because the observed antiparasitic activity cannot be readily attributed to CS inhibition and therefore warrants further investigation to elucidate the molecular target and mechanism of action of CaCS2 in trypanosomatids.

In trypanosomatid parasites, including *Trypanosoma* and *Leishmania*, mitochondrial mRNA undergoes extensive post-translational modifications characterized by the insertion and deletion of uridine (U) nucleotides [37]. In this final step of the mRNA enzymatic editing cycle, the RNA editing core complex (RECC) contains two closely related ligases, RNA editing ligase-1 and 2 (REL1 and REL2). According to studies, REL1 is primarily involved in U-deletion, while REL2 is actively linked to U-insertion [38–40]. Furthermore, REL1 is mandatory for parasites’ survival and editing, while the lack of REL2 does not block the RNA editing process, but REL1 can compensate for the editing process in vivo [41]. It’s very important to note that REL1 can take over REL2’s ligation roles, but not vice versa. So, it can be speculated that REL1 blockage may be an interesting target against parasite growth and development. Furthermore, these two proteins contain ligase motifs that share around 40% residual identity and are related to the RNA-ligase 2 of phage T4 [42]; however, they have no close homolog in the mammalian host, making it an interesting and promising target for novel drug discovery to kill parasites [43, 44].

The current study aimed to check the in vitro antiparasitic (*Leishmania amazonensis* and *Trypanosoma cruzi*) activity of a set of azobenzenoid derivative compounds, namely, PH011669, S981796, L170224, and CaCS2. Similarly, the said compounds were screened for their cytotoxicity in human cells, and their probable action mechanism in parasites using in silico tools, such as reverse docking, conventional docking, and molecular dynamics simulation studies.

## Material and Method

### Parasite and Host Cell Maintenance

*Leishmania amazonensis* promastigotes (strain IFLA/BR/1967/PH8, harboring the pIR1SAT-LUC(a)DsRed2(b) reporter gene) were propagated at 25 °C in Warren medium (pH 7.2) containing hemin, folic acid, and 10% heat-inactivated fetal bovine serum (FBS). For *Trypanosoma cruzi* (Y strain), epimastigotes were grown in Liver Infusion Tryptose (LIT) medium supplemented with 10% FBS at 28 °C. To obtain *T. cruzi* trypomastigotes, infected monolayers of LLC-MK_2_ cells (ATCC^®^ CCL-7™) were monitored, and the parasites were harvested from the supernatant. These host cells, along with J774A.1 macrophages (ATCC^®^ TIB67), were cultured in a humidified 5% CO_2_ environment at 37 °C. Specifically, RPMI 1640 (pH 7.6) was used for macrophages, while LLC-MK_2_ cells were maintained in DMEM (pH 7.2); both media were enriched with *L*-glutamine and 10% FBS.

### Antiproliferative Activity of Compounds Against*L. amazonensis*

To evaluate the effects on *L. amazonensis* promastigotes, parasites from 72-hour cultures were seeded into 96-well sterile plates at a density of 1 × 10^6^ cells/mL. The four azobenzenoid derivative compounds, namely PH011669, S981796, L170224, and CaCS2, were previously solubilized in DMSO (final concentration ≤ 1%) and were added immediately after seeding at a concentration range of 1.5–200 µM, prepared by serial two-fold dilutions. Miltefosine was used at concentration of 1.5–200 µM as a reference drug. After a 72-hour incubation period at 25 °C, parasite density was quantified using a Neubauer chamber under light microscopy. Growth inhibition was calculated relative to untreated controls, and inhibitory concentrations for 50% of the parasites (IC_50_) values were derived via non-linear regression. All assays were performed in triplicate, and results are shown as mean ± standard deviation (SD).

Macrophage infection was established by co-incubating J774A.1 cells (5 × 10^5^ cells/mL) with stationary-phase promastigotes at a 1:10 ratio in 96-well white plates. The plates were kept at 34 °C under 5% CO_2_ for 24 h to promote parasite internalization and differentiation. Following the removal of non-internalized parasites by washing, the infected monolayers were exposed to the compounds (0.1–100 µM, prepared by serial two-fold dilutions) for 48 h, using miltefosine (0.1–100 µM) as a reference drug. Parasite viability was assessed by bioluminescence using the Pierce Firefly Luc One-Step Glow Assay kit. Signals were detected with a SpectraMax L luminometer (λ_em_ = 570 nm, 1 s integration). The IC₅₀ was determined by non-linear regression based on the inhibition percentage of the treated groups versus the control [45].

### Antiproliferative Activity of Compounds Against*T. cruzi*

*T. cruzi* epimastigotes (Y strain, 96-h culture) were adjusted to 1 × 10^6^ parasites/mL and immediately treated with the test substances (1–200 µM in 1% DMSO, prepared by serial two-fold dilutions) for 96 h at 28 °C. Benznidazole (1.5–200 µM) served as the positive control. Parasite concentration was determined by direct counting in a hemocytometer. Compounds showing an IC_50_ < 100 µM against epimastigotes were selected for subsequent evaluation against trypomastigotes. For the trypomastigote stage, parasites (1 × 10^7^ cells/mL in DMEM + 10% FBS) were immediately incubated with the compounds or benznidazole (1.5–100 µM) for 24 h at 37 °C. Viability was assessed by motility using the Pizzi-Brener method under an Olympus CX31 microscope. The effective concentration for 50% of parasites (EC_50_) and IC_50_ values were calculated from at least three independent experiments using non-linear regression.

### Evaluation of the Cytotoxicity of Substances

The cytotoxic potential of the compounds was tested against J774A.1 (5.0 × 10^5^ cells/mL) and LLC-MK_2_ (2.5 × 10^5^ cells/mL) lineages. Cells were initially cultured for 24 h (37 °C, 5% CO_2_) before the addition of the substances (15.625–1000 µM, prepared by serial two-fold dilutions). After incubation periods of 48 h (macrophages) and 96 h (LLC-MK_2_), cell viability was measured via the MTT reduction assay. Briefly, after PBS washing, cells were incubated with 2 mg/mL MTT for 4 h [46]. Formazan crystals were dissolved in DMSO, and absorbance was recorded at 570 nm (Power Wave XS, BioTek). The cytotoxic concentration for 50% of the cells (CC_50_) values were estimated through non-linear regression analysis of data from three independent assays.

Since only CaCS2 showed interesting anti-parasitic potential against *Trypanosoma* and *Leishmania*, and remained non-cytotoxic to human cell lines. In further computational analysis, only CaCS2 was considered.

### In Silico Compound Retrieval and Target Prediction

The 2D structures of the azobenzenoid derivatives used in this work were downloaded from the PubChem database for CaCS2 (CID 79465), followed by 4,060,885, 5,282,251, and 135,406,014 in .sdf format. Using Open Babel (version 3.1.1), a library was made, thereby compiling all compounds in a single file, and the protonation states were adjusted to pH 7.0; this level did not alter the molecular connectivity or covalent structure of the compounds; it only adjusted the hydrogenation state and formal charges of ionizable functional groups where applicable [47]. According to the BRENDA database, the optimal pH for REL1 is between 7.0 and 7.5. Generally, enzymes at pH 7.0 work well, approximate physiological conditions, and provide chemically relevant protonation states for the subsequent target prediction and molecular docking analysis [48]. Lastly, the retrieved structures were converted to 3D format and used for the subsequent docking studies. It is quite interesting to mention that these compounds were previously reported as inhibitors of the enzyme chorismate synthase [33] and show significant activity against the fungus *Paracoccidioides brasiliensis* [36].

The target prediction was evaluated using Sea Search Server [49]where the results were filtered by the following criteria: Be present in *Leishmania* or *Trypanosoma* genomes and have a significant SEA *p*-value and MaxTC score. Where the similarity ensemble approach (SEA) *p*-value measures the statistical significance of the global set-wise chemical similarity between the query compound (ligand) and annotated target ligands. Lower/more negative *p*-values indicate much more similarity of the query compound with the annotated ligands. Similarly, MaxTC (Maximum Tanimoto coefficient) score ranging from 0 to 1 quantifies a single relative nearest-neighbor structural similarity to the known ligand for that annotated target ligand [50].

### Structure Modeling

**Fig. 1:**
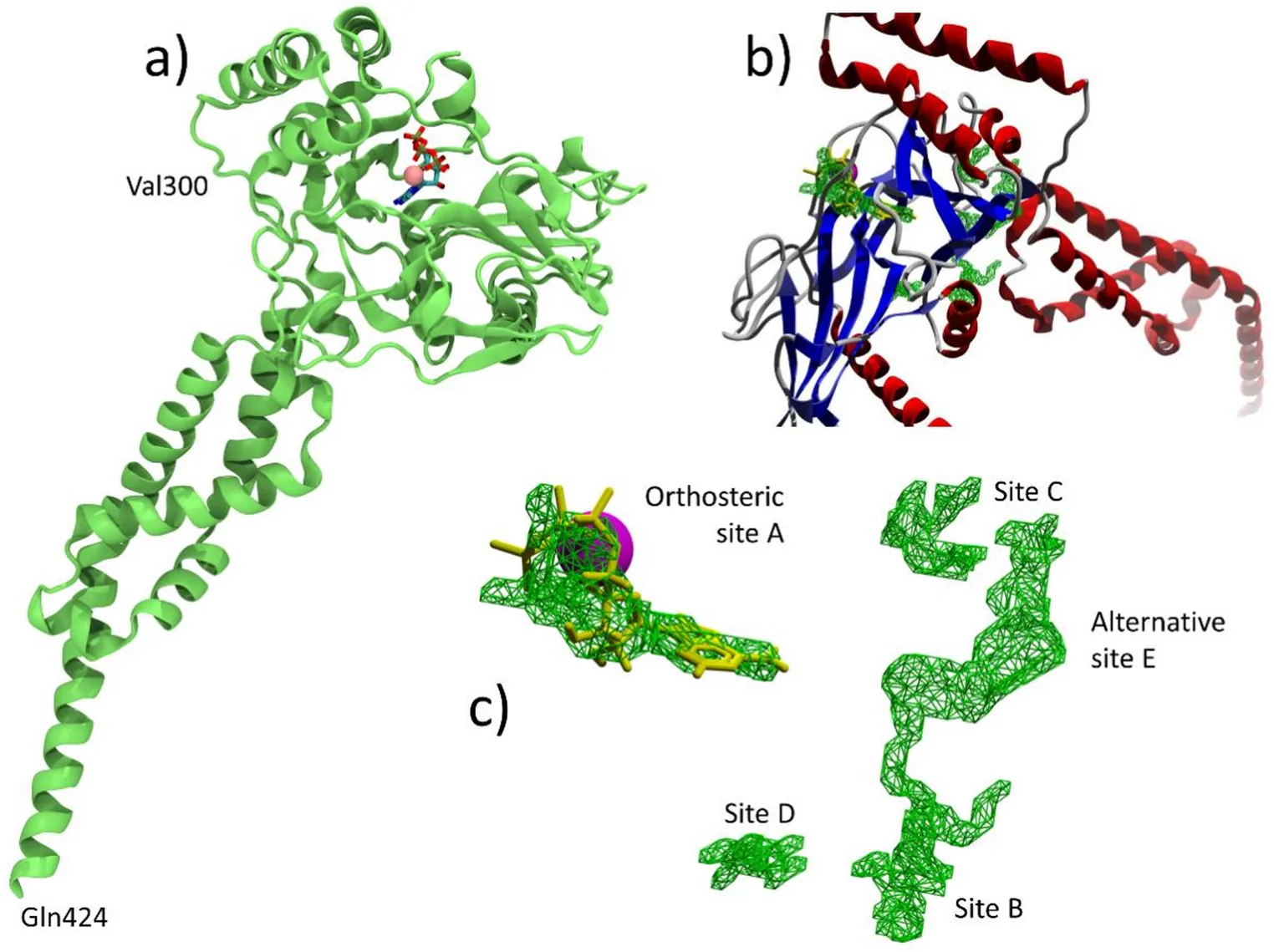
Structural representation and docking sites identification of REL1, a possible drug target in *Trypanosoma* and *Leishmania*. Where **a** shows a ribbon 3-dimensional structure, with ATP and Mg^2+^ cofactor bound at the orthosteric site. Similarly, **b** showing 3-dimensional structure with α-helices (red), β-sheets (blue), loops (grey), and binding cavities are highlighted (green mesh). Furthermore, **c** highlights surface representation of REL1, illustrating the orthosteric site (A) and multiple predicted alternative binding cavities (B-E), among which alternative site E is a bigger cavity. Cavities and images (b, c) were generated and visualized by the Molegro-6.0 software package

The AlphaFold model structures of REL1, which were suggested by the SEA predictions as possible targets for both *Leishmania braziliensis* (Uniprot: A4H382) and *Trypanosoma cruzi* (Uniprot: Q6T434), were downloaded from Uniprot in *.pdb format. These structures were in the inactive Apo form; the active form of the enzyme, bonded to ATP and Mg^+^ cofactors, was modeled by geometric docking using the crystal structure of *Trypanosoma brucei* RNA Editing Ligase 1 (pdb id: 1xdn) as a template [51] (Fig. 1).

Using the Coot package, AlphaFold-predicted structures *L. braziliensis* and *T. cruzi* were superimposed onto the crystal structure of *T. brucei* REL1 editing ligase 1 (REL1) containing ATP and Mg^2+^ cofactors. Similarly, both AlphaFold models exhibited a high degree of structural similarity with the reference REL1 structure, particularly in the conserved catalytic regions. The ATP-binding site and Mg^2+^-coordinating environment were also structurally conserved in both predicted models and were therefore retained as a reference for the subsequent structural and molecular docking analysis.

### Molecular Docking Simulations

#### Guided Docking Using AutoDock Vina

The Vina software package implemented within the PyRx 1.0 graphical interface [52] was also used in the first guided docking attempt. The docking protocol used default settings with box sizes of 18, 18, and 18 along the *x*, *y*, and *z* axes, respectively, centered on the orthosteric site cavity. For the alternative site cavity, the box dimensions were 20, 25, and 20 along the *x*, *y*, and *z* axes, respectively. The exhaustiveness parameter was set to 20 in all simulations. Four attempts were carried out at both sites; the binding scores were noted, and the resulting poses were visualized using UCSF Chimera 1.13.1 software.

#### Binding Site Prediction

Since no crystallographic structures of REL1 in the presence of ligands were found in PDB, the likely binding sites on the protein were identified by blind docking. Initially, the structures in the active form (bound to ATP and Mg^2+^ cofactors) had their cavities identified using the Molegro v 6.0 program. Among the predicted/suggested binding sites, two major cavities, designated sites A and E, were identified, along with three smaller cavities (sites B, C and D). Of the two larger pockets site A corresponded to the orthosteric ATP/Mg^2+^ cofactor-binding region, whereas, site E represented an alternative cavity located on the opposite side of the protein (Fig. 1).

To carry out the docking simulations, four REL1 structures were modeled, i.e., two for *Leishmania* and two for *Trypanosoma*, with and without ATP/Mg^2+^ cofactors. Blind docking was performed using species-specific docking grids. For example, in the case of *Leishamnia*-REL1 mode, the grid was defined by center coordinates of 4, 5, 0 along *x*, *y*, and *z*, axes respectively, with a grid size of 70, 75, and 60. Similarly, for the *Trypanosoma*-REL1 model, the grid was centered at 2, 3, and 0 for *x*, *y*, and *z*, with a grid size of 66, 55, and 60, respectively. Following these docking parameters, 50 blind docking attempts were performed for *Leishmania*’s REL1 and *Trypanosoma* REL1, both in the apo form (without ATP/Mg^2+^ at the orthosteric site). The blind docking was performed using Vina software package implemented within the PyRx 1.0 graphical interface [52]. The objective of these docking simulations was to check whether the ligand CaCS2 may bind in some of the cavities identified by the Molegro-6.0 program and in which cavity the binding is more frequent.

Similarly, in the next step, we carried out guided docking (4 attempts), thereby considering two main cavities i.e., the orthosteric site (a) and the alternative site (e), for both *Leishmania* and *Trypanosoma* structures (with and without ATP/Mg^2+^). We employed different docking software packages to understand and verify the frequency and drug-like properties of the CaCS2 at orthosteric and alternative binding sites within the REL1 enzyme.

#### Guided docking using AutoDock

AutoDock v. 4.2.3, implemented in the PyRx 1.0 graphical interface, was used to validate our findings. The program used the default parameters, grid spacing of 0.375, and box dimensions of 50, 50, and 50 along the *x*, *y*, and *z* axes, respectively, centered on the orthosteric site cavity. For the alternative site cavity, the box dimensions were 60, 60, and 60 along the *x*, *y*, and *z* axes, respectively. The grid spacing was set to 0.375 in all simulations. Four attempts were carried out at both sites; the binding scores were noted, and the resulting poses were visualized using UCSF Chimera 1.13.1 software.

#### Guided Docking Using Molegro

The Molegro-6.0 docking software was also used to further validate our findings. This program used the Iterated Simplex algorithm for search, and the Moldock Score algorithm for ranking. The radius of the search sphere was set to 9 Å in both sites (orthosteric and alternative). In the same way, each docking attempt was repeated four times to confirm drug-likeness in each binding site. The binding scores were noted, and the resulting poses were visualized using UCSF Chimera 1.13.1 software.

Since each program generates scores with different scales, whose numerical values cannot be directly compared, a mean relative score for CaCS2 binding at each site was calculated according to the following relation, to make them comparable to one another:

1 $$\:Mean\:Relative\:Score=\:\frac{1}{3}\left(\frac{Vina}{{Vina}_{max}}+\frac{AutoDock}{{AutoDock}_{max}}+\frac{Molegro}{{Molegro}_{max}}\right)$$

where *Vina*_*max*_, *AutoDock*_*max*_, and *Molegro*_*max*_ correspond to the CaCS2 score at a given site, to which the maximal numerical value was attributed by each program.

### Molecular Dynamics (MD) Simulation

To further validate CaCS2 binding at either site of REL1 in *Trypanosoma* and *Leishmania*, molecular dynamics (MD) simulations were carried out using the GROMACS version 2018 x series molecular dynamics package following Lemkul et al. [53]. The best docking poses were selected, and ligand parameters were prepared using the CGenFF server [54]. Similarly, ligands and protein GROMACS-compatible topology were prepared using the CHARMM36 force field, and the protein-ligand complex was constructed by merging their coordinates and updating the system topology. The complex was then placed in a cubic box with a 1.5 nm distance from the outermost surface of the protein, followed by the addition of Na^+^ and Cl^−^ ions to neutralize the system charges [55]. Energy minimization was performed by applying the steepest descent algorithm to remove steric clashes, after which the system was equilibrated under NVT (300 K) and NPT (1 bar) conditions with position restraints applied to allow solvent relaxation [56]. Finally, an undertrained production simulation of 100 ns was conducted for each complex. The trajectories were processed to correct periodic boundary conditions and centered before analysis. The complex behavior properties, including root mean square deviation (RMSD), root mean square fluctuation (RMSF), radius of gyration, and contact frequency between protein and ligand atoms, were obtained using VMD software version 1.9.3 [57]. The long-time MD took place on a cluster Dell AMD Epyc 7662 / nVIDIA Tesla A100 at CENAPAD/SP (proj. 520). The statistics of the data were properly analyzed through the *t*-test at a 0.05 level of significance.

## Results

### In Vitro Antiparasitic Assay

The antiparasitic potential of the tested compounds was evaluated against different life stages of *L. amazonensis* (promastigotes and amastigotes) and *T. cruzi* (epimastigotes and trypomastigotes), along with cytotoxicity evaluation in mammalian cell lines (J774A.1 and LLC-MK_2_) (Table 1).

Among the evaluated compounds, PH011669 and S981796 showed no significant activity against promastigote forms of *L. amazonensis* and epimastigote forms of *T. cruzi* (IC_50_ > 200 µM). However, both compounds exhibited pronounced activity against intracellular amastigotes of *L. amazonensis*, with IC_50_ values of 9.97 ± 0.10 µM and 7.79 ± 0.26 µM, respectively. These results are particularly relevant given that the amastigote stage represents the clinically relevant form of the parasite. In addition, both compounds displayed high selectivity indices (SI = 54.08 for PH011669 and 44.79 for S981796), which can be attributed to their low cytotoxicity in mammalian cells (CC_50_ > 300 µM), indicating a favorable therapeutic window.

L170224 exhibited a distinct activity profile. This compound was active against both promastigote (IC_50_ = 13.60 ± 0.98 µM; SI = 39.34) and amastigote forms (IC_50_ = 9.81 ± 0.38 µM; SI = 54.54) of *L. amazonensis*. However, no activity was observed against *T. cruzi* epimastigotes (IC_50_ > 200 µM). Despite this limitation, its high selectivity indices reinforce its potential as a selective antileishmanial agent.

In contrast to the other compounds, CaCS2 (CID-79465) demonstrated a broad-spectrum antiparasitic profile, showing consistent activity across all evaluated parasite stages. For *L. amazonensis*, CaCS2 exhibited IC_50_ values of 9.30 ± 0.80 µM against promastigotes (SI = 60.28) and 11.60 ± 1.70 µM against amastigotes (SI = 36.90). For *T. cruzi*, the compound remained active against both epimastigotes (IC_50_ = 12.90 ± 1.40 µM; SI = 52.21) and trypomastigotes (EC_50_ = 22.10 ± 2.90 µM; SI = 30.48). Importantly, CaCS2 exhibited low cytotoxicity toward mammalian cells, with CC₅₀ values of 428.0 ± 7.1 µM (J774A.1) and 673.5 ± 8.4 µM (LLC-MK2), further supporting its favorable selectivity profile.

When compared to reference drugs, miltefosine showed higher potency against amastigotes (IC_50_ = 2.95 ± 0.15 µM), although with lower selectivity (SI = 17.35), while benznidazole presented expected activity against *T. cruzi* forms but with lower selectivity indices (SI = 4.13).

Overall, although PH011669, S981796 and L170224 demonstrated selective activity against *L. amazonensis*, their lack of activity against *T. cruzi* limits their broader applicability. In contrast, CaCS2 emerged as the most promising compound due to its balanced potency, broad-spectrum antiparasitic activity, and low cytotoxicity, supporting its selection for further mechanistic and in vivo studies.

**Table 1:** Antiproliferative activity of inhibitors against the protozoa *Leishmania amazonensis* and *Trypanosoma cruzi* and cytotoxicity in J774A.1 and LLC-MK_2_ cells

Compounds	J774A.1	Leishmania amazonensis				LLC-MK_2_	Trypanosoma cruzi			
		Promastigotes		Amastigotes			Epimastigotes		Trypomastigotes	
	CC_50_ ± SD (µM)	IC_50_ ± SD (µM)	SI	IC_50_ ± SD (µM)	SI	CC_50_ ± SD (µM)	IC_50_ ± SD (µM)	SI	EC_50_ ± SD (µM)	SI
PH011669	539.21 ± 25.07	> 200	< 2.70	9.97 ± 0.10	54.08	617.55 ± 53.63	> 200	< 3.09	NT	ND
S981796	348.93 ± 33.43	> 200	< 1.74	7.79 ± 0.26	44.79	675.50 ± 20.78	> 200	< 3.38	NT	ND
L170224	535.04 ± 18.96	13.60 ± 0.98	39.34	9.81 ± 0.38	54.54	635.48 ± 33.98	> 200	< 3.18	NT	ND
CaCS2	428.00 ± 7.10	9.30 ± 0.80	60.28	11.60 ± 1.70	36.90	673.50 ± 8.40	12.90 ± 1.40	52.21	22.10 ± 2.90	30.48
Miltefosine	51.19 ± 4.23	23.61 ± 1.24	2.17	2.95 ± 0.15	17.35	NT	NT	ND	NT	ND
Benznidazole	NT	NT	ND	NT	ND	30.79 ± 3.39	6.43 ± 0.44	4.79	7.45 ± 0.04	4.13

CC_50_ = cytotoxic concentration for 50% of cells. IC_50_ = inhibitory concentration for 50% of protozoa. EC_50_ = effective concentration against 50% of protozoa. SD = standard deviation. SI = selectivity index (CC_50_/IC_50_ or EC_50_). ND = not determined. NT = Not tested.

### In Silico Target Prediction

Target prediction using the SEA server showed that compound CaCS2 (CID-79465) may interact with RNA-editing ligase 1 (REL1), with a *p*-value of 1.761 × 10^−12^ and a MaxTC of 0.31. More interestingly, the homologs of REL1 are entirely present in *Trypanosoma*, as well as in *Leishmania*, but not in human beings [58]. It was quite an eye-catching point that REL1 is a key enzyme mandatory for the survival of both microbes, due to its crucial role in mRNA post-transcriptional modification. According to our in vitro findings, CID-79,465 inhibited the growth and development (causing death) of both microbes, further strengthening our hypothesis that CID-79,465 could inhibit the REL1 enzymatic activity.

### Docking Simulations

After 50 blind docking attempts using *Leishmania-*REL1 in the apo form, we found 27 poses at the ATP orthosteric site (site A), which assumed completely random conformations despite being within the cavity. Similarly, we found 23 drug-like poses at the alternative site (cavity E). Furthermore, another 50 docking attempts were carried out for the *Trypanosoma-*REL1 structure in the apo form, showing that CaCS2 also binds only at the orthosteric site (cavity A), but at random conformations (Supplementary file Table **S1**). Since the results suggested the presence of an alternative site for CaCS2 in the REL1 structure, it is reasonable to consider that this could be a putative alternative binding sitewhere CaCS2 could bind and regulate the enzyme activity. Therefore, in the next step, simulations of guided docking were performed on the REL1 structures of *Leishmania* and *Trypanosoma* in both cavities A (orthosteric site) and E (alternative site). Due to the small sizes of cavities B and C, the ligand did not visit them during the 100 blind docking simulations (50 attempts in each structure); therefore, they were disregarded from the subsequent guided docking analyses.

The guided docking of CaCS2 at the two major cavities in *Leishmania* and *Trypanosoma* was evaluated using three different docking programs, where the results showed consistent site-dependent variations (Table 2). The redocking ATP at its (orthosteric) site yielded the best relative score in REL1 for both species. Since ATP is the primary substrate of this enzyme, it was expected that it would exhibit a very high score. Similarly, the binding of CaCS2 at the orthosteric site showed the second-best scores in REL1 of both species, and the binding of CaCS2 at the alternative site showed the lowest scores (Table 2). From this guided docking, it can be concluded that CaCS2 binds better at the orthosteric site than at the alternative site.

**Table 2:** Mean relative scores from guided docking attempts of CaCS2 across REL1 binding cavities using different software packages

Guided docking using different programs		
Protozoa	Target sites	Mean relative score
	CaCS2-alternative	0.83
*Leishmania*	CaCS2-orthosteric	0.87
	ATP-orthosteric	0.88
	CaCS2-alternative	0.69
*Trypanosoma*	CaCS2-orthosteric	0.87
	ATP-orthosteric	0.90

### **2D Interactions**

**Fig. 2:**
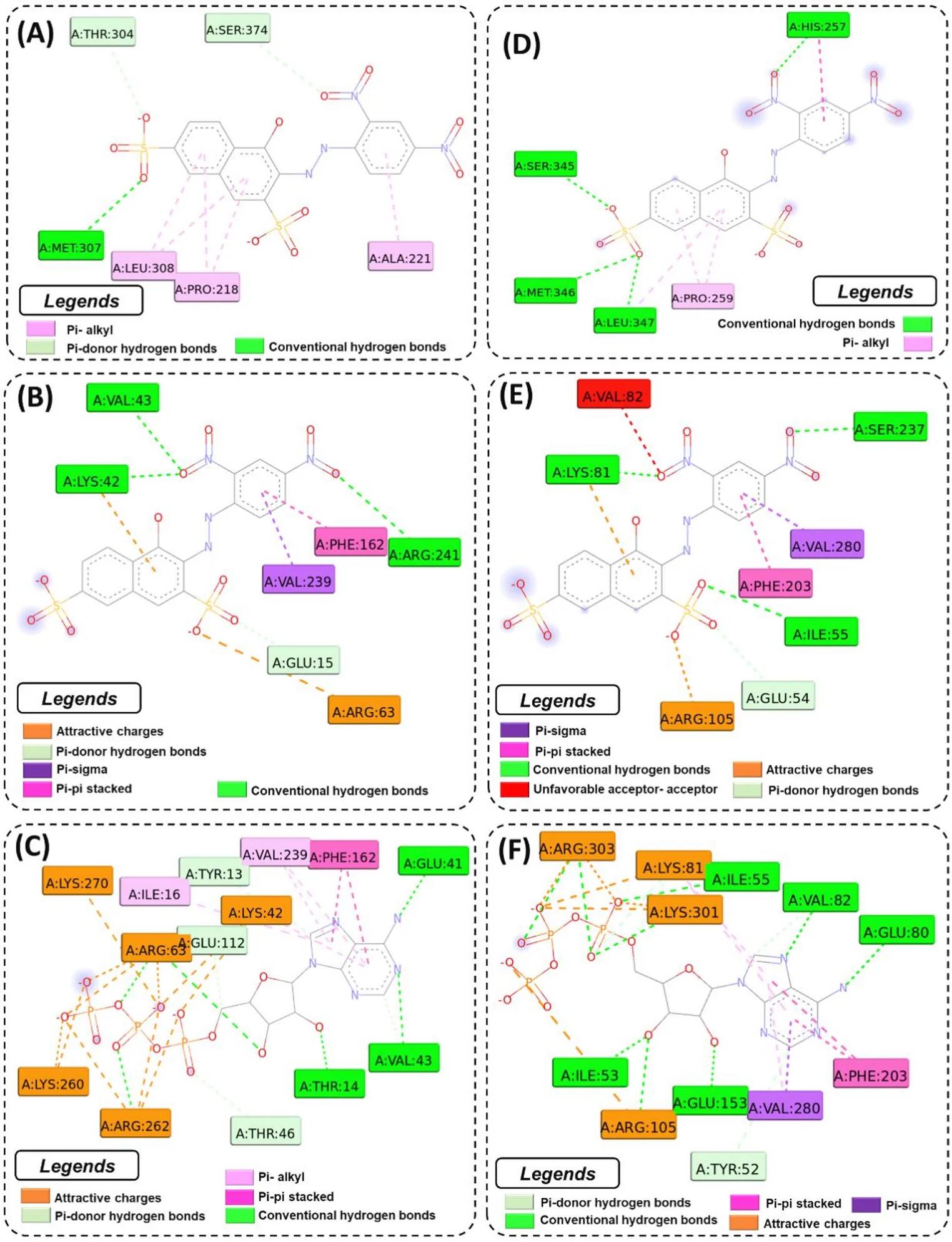
Two-dimensional interaction profiles of CaCS2 with REL1 in *Leishmania* (**A–C**) and *Trypanosoma* (**D–F**). More specifically, showing **A** the CaCS2 binding mode at the alternative site; **B** the CaCS2 binding at the orthosteric site, and **C** the ATP binding at the orthosteric site in *Leishmania*. Similarly, the binding mode of CaCS2 at the alternative site (**D**), the CaCS2 binding at the orthosteric site (**E**), and the ATP binding at the orthosteric site (**C**) of *Trypanosoma*, respectively. The docking poses were visualized in Discovery Studio

The CaCS2 binds at an alternative site of REL1 in *Leishmania* (Fig. 2A), exhibiting stable interaction through a single hydrogen bond to Met307, followed by pi-donor hydrogen bonds with Thr302 and Ser374, respectively. Similarly, other interactions include hydrophobic (Pi-alkyl) interactions with Ala221 and double interactions with Pro218 and Leu308. In contrast, CaCS2 binds at the orthosteric site of REL1 in *Leishmania*, indicating promising interactions with key amino acids (Fig. 2B). Among these, three hydrogen bonds with Lys42, Val43, and Arg241, as well as a single pi-pi stacking interaction with Phe162 and a pi-sigma interaction with Val239, were also noted. Additionally, attractive charges (electrostatic interactions) with Arg63 and Lys42, a pi-donor hydrogen bond with Glu15, were also formed. Furthermore, ATP (cofactor) at its orthosteric site (Fig. 2C) showed a total of six hydrogen bonds, Arg63 forming double bonds, followed by a single bond with Arg262, Thr14, Glu41, Val43, followed by three attractive charges with Arg262 and Arg63, double bonds with Lys42 and Lys260, while a single attractive charge bond with Lys270. Similarly, the presence of two pi-pi stacked interactions with Phe162, followed by two pi-alkyl bonds with Val239 and a single pi-donor hydrogen bond with Ile16, followed by three pi-donor hydrogen bonds with Tyr13, Thr46, and Glu112, is also evident.

Similarly, CaCS2 also binds at the alternative site of REL1 in *Trypanosoma*, exhibiting several key hydrogen bonds with key residues, including His257, followed by Ser345, Met346, and Leu347. Similarly, it also formed double pi-alkyl bonds with Pro259 and two single interactions with His257 and Leu347 **(**Fig. 2D**)**. Furthermore, CaCS2 binds at REL1’s orthosteric site, exhibiting three conventional hydrogen bonds with key amino acids including Ile55, Lys81 and Ser237 (Fig. 2E). Other interactions involved a single pi-sigma with Val280, a single pi-pi stacked with Phe203, a single attractive charge with Glu54, and an unfavorable acceptor-acceptor bond with Val82. Similarly, ATP (cofactor) at its orthosteric site of REL1 in *Trypanosoma* also showed extensive interactions (Fig. 2F). Among others, double conventional hydrogen bonds were formed with Arg303, followed by single hydrogen bonds with Ile53, Ile55, Glu80, Val82, Arg105, Glu153, and Lys301, respectively. Moreover, double attractive charges were formed with Lys 301 and Lys303, followed by single attractive charges with Lys81 and Arg105. Similarly, a single pi-donor hydrogen bond with Tyr52, followed by a double pi-pi stacked interaction with Phe203 and Lys81, followed by a double pi-sigma with Val280. It was interesting to note that these interactions collectively stabilize the ligand with the binding site, indicating a favorable binding orientation.

### MD Simulations

The docking simulations suggested that the ATP substrate binds to the orthosteric site with the highest score, followed by binding of CaCS2 at the same site, and binding of CaCS2 at the alternative site, which exhibited the lowest relative score. However, the mean relative scores were very close, making it impossible to obtain a clear view of binding preferences. Therefore, equilibrium molecular dynamics simulations were performed to verify the stability of the ligands at the evaluated sites.

**Fig. 3:**
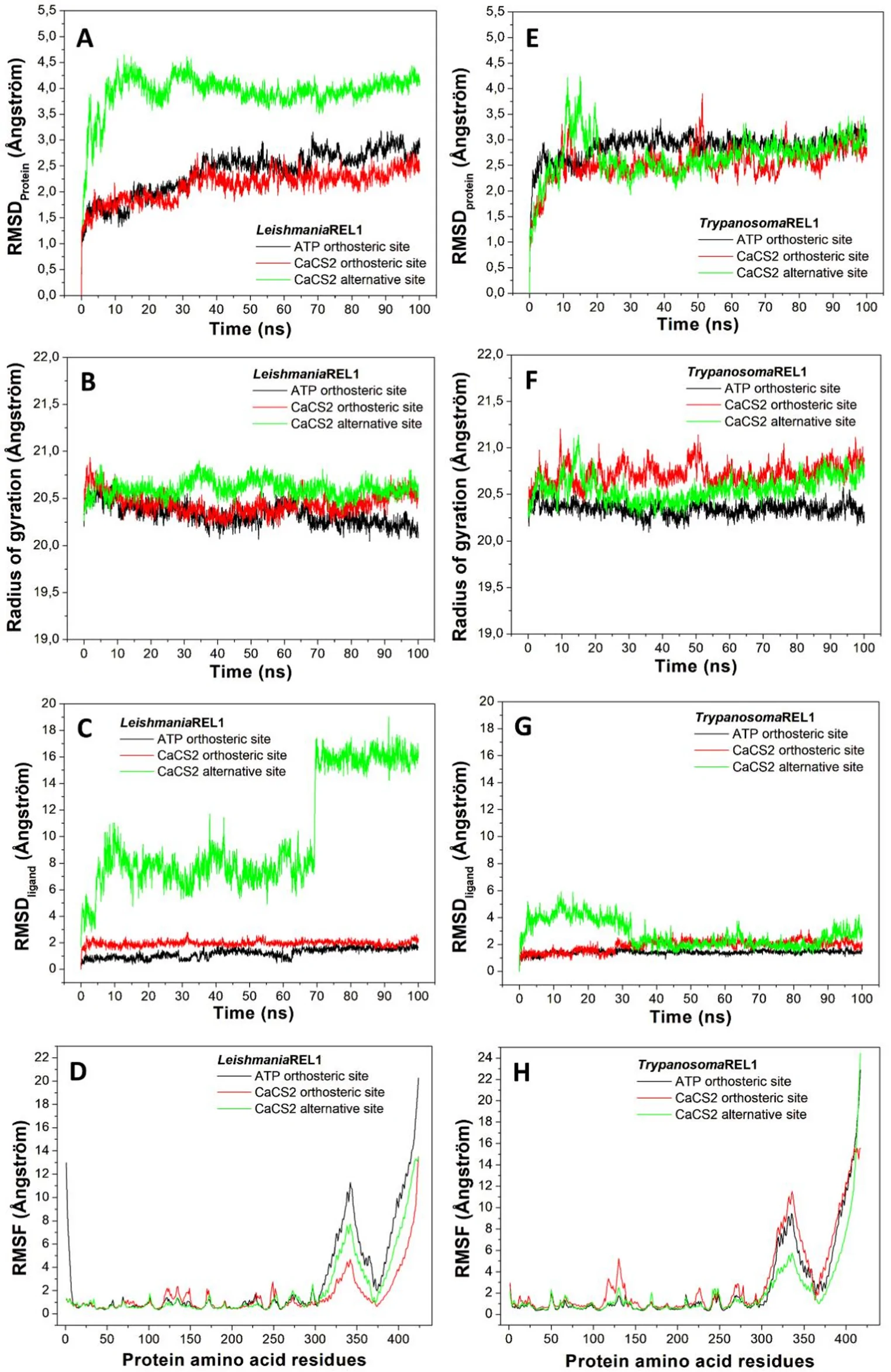
Molecular dynamics simulation parameters for *Leishmania-*REL1 (**A**–**D**) and *Trypanosoma-*REL1 (**E**–**H**). Root mean square deviation of REL1 main chain atoms (**A** and **E**). Radius of gyration of REL1 main chain atoms (**B** and **F**). Root mean square deviation of the ligand atoms (**C** and **G**). Root mean square fluctuation of protein Cα atoms

The root-mean-square deviation (RMSD) of the protein backbone indicates that the REL1 complexes reached thermodynamic equilibrium after 40 ns (Fig. 3A and E) in all systems. In both *Leishmania* and *Trypanosoma* REL1, the systems with CaCS2 bound to the alternative site showed the lowest stability. The systems with ATP and CaCS2 bound to the orthosteric site demonstrated similar protein stability.

The radius of gyration (*Rg*) of all systems has a similar behavior around 20.5 ± 0.5 Å (Fig. 3B and F), indicating that the presence of the ligands does not allow the protein to unfold, a fact that provides clues about the mechanism by which the compounds inhibit the protein.

The analysis of the ligand’s RMSD indicates that CaCS2 at the alternative site is unstable, eventually leaving this site permanently at the beginning of the simulation in *Leishmania-*REL1. Similarly, in the *Trypanosoma*-REL1 system, the CaCS2 undocks from the alternative site at the beginning of the simulation and then returns to it after 35 ns (Fig. 3C and G). On the other hand, the binding of CaCS2 at the orthosteric site remained stable throughout the simulation in REL1 for both species.

The MD simulations enabled the identification of flexible regions within the protein by analyzing the root-mean-square fluctuation (RMSF) of the Cα atoms for each residue in the presence and absence of ligands (Fig. 3D and H). For either system, RMSF was calculated for the last 20 ns, when the proteins were in their equilibrium state. It was observed that the protein maintains overall structural stability, with the majority of residues exhibiting low fluctuation of the Cα atoms. This confirms the observation from the analysis of the radius of gyration, in which the ligands did not allow the protein to unfold. The greatest variation observed from residue 300 is due to five accessory alpha-helices (Fig. 1a) with a high degree of freedom of movement. However, these helices are located a great distance from the orthosteric site and very close to the alternative site. Due to this, the mobility of these helices may have interfered with the stability of CaCS2 at the alternative site in REL1 of both species.

### Contact Frequency

From MD simulation trajectories, it was possible to calculate the contact frequency between key amino acids and ligands within 4.0 Å distance in the last 20 ns of simulation, a time when the protein and ligands reach thermodynamic equilibrium (Supplementary file, Tables S3 and S4). Frequencies greater than 0.80 and/or complete occupancy 1.00, highlighting their critical role in ligand stabilization. This suggests a more stable interaction, realizing an interesting candidate to inhibit the functional integrity.

In the orthosteric site of *Leishmania-*REL1, nine key amino acid residues were identified that bind to both ATP and CaCS2, as follows: Ile16, Glu41, Lys42, Val43, Asn47, Arg63, Phe162, Val239, and Lys260. If we also consider the exclusive contacts that each of these ligands makes with the protein, this number increases even more. On the other hand, the binding of CaCS2 at the alternative site interacts only with a total of seven key residues: Gly226, Asn227, Leu308, Asn372, Thr373, Ser374, and Arg378, in smaller amounts than the orthosteric site (Supplementary file, Tables S3).

For the *Trypanosoma-*REL1, the ATP and CaCS2 ligands also bind to nine key residues in the orthosteric site: Tyr52, Ile55, Lys81, Arg105, Glu153, Phe203, Val280, Arg282, and Ile299. Again, the exclusive contacts for each ligand are not included. Comparatively, the CaCS2 binds to the alternative site with a total of six key residues, in smaller amounts than seen in the orthosteric site (Supplementary file, Tables S4).

## Discussion

In previous studies, our research group identified the compound disodium 2-(2,4-dinitrophenylazo)-1-hydroxynaphthalene-3,6-disulphonate (CaCS2) as an inhibitor of the chorismate synthase enzyme from the pathogenic fungus *Paracoccidioides brasiliensis*, which demonstrated significant antifungal activity [59]. In other studies, with Apicomplexan parasites (*Toxoplasma* and *Plasmodium*), organisms that also express the Chorismate Synthase from their genome, the compound CaCS2 also demonstrated some in vitro inhibitory activity (unpublished results).

However, in the current study, in vitro findings demonstrate the antiproliferative activities of CaCS2 against *L. amazonensis* and *T. cruzi*, while it remained non-cytotoxic against macrophages J774A.1 and epithelial cells LLCMK_2_. Our recent findings are consistent with our previous results with CaCS2; however, parasites of the family Trypanosomatidae do not express the enzyme chorismate synthase, which raises questions about how CaCS2 might be acting on these organisms. Finding a suitable drug candidate for *Leishmania* is a challenge; however, different research groups have made their efforts. For example, Otero et al. [60] synthesized four tricosan-based hybrid compounds, which showed interesting anti*-leishmanial* activity with negligible toxicity. Another study conducted by Al Nasr et al. [61], also investigated the anti-parasitic activities of 3-hydroxy-2-naphtholic hydrazide 2a via in vitro and in vivo approaches.

Our experimental findings demonstrated that CaCS2 and some azobenzenoid derivatives showed an antiproliferative effect on trypanosomatid parasites without being toxic to mammalian cell lines (Table 1); however, the mechanism of action of these compounds remained unknown. The SEA predictions server is a possible way to get an indication of the probable mechanism by means of reverse docking approach. In this methodology, the 1D structure of the query molecule is subjected to the analysis of the database. If the query molecule resembles a given ligand (ligand X) described in the database, likely, the query molecule will also bind to the same target described for ligand X. Among the possible targets suggested by Sea Predictions is the RNA-editing ligase 1 enzyme (REL1, Uniprot ids: A4H382 and Q6T434), an enzyme that belongs to the RNA ligase family (E.C. 6.5.1.3). In trypanosomatid parasites, including *Trypanosoma* and *Leishmania*, mitochondrial mRNA generally undergoes extensive post-transcriptional editing and modifications [62]. This involves uridine insertion and deletion, in an ATP-dependent way, where RNA ligases such as REL1 and RNE2 are involved in RNA ligases [44, 63]. Among these, REL1 is essential for parasite survival and has no close homologs that have been identified in mammals [64].

Following these predictions, the REL1 structures of *L. amazonensis* and *T. cruzi*, modelled by the AlphaFold server and made available in the Uniprot database, were obtained and modelled again by geometric docking for the forms bound to ATP and the magnesium cofactor. Although the active site in the REL1 was previously well reported, the search for cavities using the Molegro program and also by blind docking, suggested an alternative binding site, and hence we proceeded with a couple of docking attempts. We found that CaCS2 docking was good for both the orthosteric and alternative sites with drug-like poses. Our efforts to verify the possibility of CaCS2 acting as an inhibitory ligand of REL1 have highlighted challenges associated with targeting conserved catalytic sites, supporting the exploration of alternative binding regions to improve ligand accommodation and selectivity [65]. Since the active form of REL1 contains an ATP cofactor at the orthosteric site, our group has previously reported that CaCS2 at pH 7.0 has a partition coefficient (*log*P) of -1.25 and a negative net charge (-2) [34]. These negative charges seem to be essential for the ligand’s stability in the active site (orthosteric site), as ATP does [66].

However, the possibility of CaCS2 binding to an alternative site and acting as a negative allosteric effector of the enzyme was suggested by docking simulations. To verify this hypothesis, we performed molecular dynamics (MD) simulations with CaCS2 at both sites to evaluate the stability and persistence of ligand-protein complexes over time. MD simulation is a computational approach, that enables the characterization of the time-dependent behavior of molecular systems by modeling the physical movement of atoms and dynamic interactions between a ligand and its macromolecules/protein target [67].

The root mean square deviations (RMSD) of REL1 main chain atoms with the CaCS2 bond at the orthosteric site of both species, i.e., *Leishmania* and *Trypanosoma *(Fig. 3A and E), remain stable; however, at the alternative site, the structure remained unstable with high oscillation. The stable behaviour of CaCS2 at the orthosteric site reflects the involvement of multiple residues in binding, providing greater stability and drug-like behaviour, as the protein remains stable with negligible oscillations [68]. The RMSD of ligand atoms also showed that the interaction of CaCS2 with the orthosteric site is more stable compared to the interaction with the alternative site, in which CaCS2 detached and diffused into the solvent in *Trypanosoma-*REL1 (Fig. 3C). Similarly, same consequences occur with CaCS2 at the alternate site of *Leishmania-*REL1, where the ligand detached from the alternate site at the beginning of the simulation but re-bound after 35 ns (Fig. 3G). It’s quite obvious that the ligand leaves its binding site at the start of the MD simulation due to various reasons, for example, the lack of stable thermodynamic interactions, the starting pose is relaxed, and/or electrostatic repulsion occurs with the protein residues [69, 70]. The possible reasons for the high fluctuations of CaCS2 at the alternative sites are ligand accommodation, minimum residues for binding (supplementary file Tables S3 and S4), abrupt conformational changes, and drastic modifications, which most likely represent the ligands’ capability to adapt to the binding pocket [65, 71]. On the other hand, the binding of CaCS2 to the orthosteric site showed greater stability in REL1 of both species, resembling the behaviour of the ATP ligand (Fig. 3A, E, C and G), as well as sharing contact with many of the same key residues in this site (supplementary file Tables S3 and S4).

The radius of gyration (*Rg*), which measures the average quadratic distance between a group of atoms and their shared centre of gravity, was also used to assess the stability of the formations. In the current study, very low fluctuations were observed in either complex, and the protein unfolding was not seen for any of the ligands in any sites (Fig. 3B and F). The root mean square fluctuation of Cα (RMSF) also proved stable for the globular region of the protein (residues 1 to 300) in all complexes evaluated (Fig. 3D and H). Together, the *Rg* and RMSF analyses suggest that CaCS2 binding does not destabilize the structure of REL1, suggesting, along with the RMSD data, that the mechanism by which CaCS2 may eventually inhibit REL1 would be through competition with ATP and not as a negative allosteric effect by binding to an alternative site.

## Conclusion

The current study demonstrates the promising antiparasitic potential of azobenzenoid derivatives, especially the CaCS2 as a dual targeting inhibitor of *Trypanosoma cruzi* and *Leishmania amazonensis* parasites. The combined in vitro and in silico findings indicate that CaCS2 exhibits selective biological activities against clinically relevant parasitic forms of both pathogens, while maintaining low cytotoxicity towards mammalian cells (J774A.1 and LLC-MK_2_). Similarly, in silico target prediction revealed REL1 as a plausible molecular target by CaCS2. A series of computational simulations, including docking attempts and equilibrium molecular dynamics, suggests that the orthosteric binding site shows an interesting drug-like interaction profile than the alternative site. Furthermore, detailed molecular dynamics simulations confirmed that CaCS2 remained the best drug candidate at the orthosteric site (maintaining stable parameters). The observed antiparasitic activities caused by CaCS2 are a promising lead scaffold for the development of novel broad-spectrum antiparasitic agents and support further mechanistic and in vivo investigations to validate therapeutic potential. However, further enzyme-specific assays related to REL1 inhibition are recommended to further clarify CaCS2’s potential inhibition of parasites.

## Supplementary Information

Below is the link to the electronic supplementary material.

Supplementary Material 1.

## Data Availability

No datasets were generated or analysed during the current study.
