# Supplementary material for: Integrated Experimental and Computational Investigation of Azobenzenoid Derivatives Against Trypanosomatid: RNA Editing Ligase 1 as a Potential Molecular Target
Source: Acta Parasitol. 2026 Sep 7;71(5):201. doi: 10.1007/s11686-026-01391-1 (PMC13550046; doi:10.1007/s11686-026-01391-1)
Supplement: Supplementary file 1 [file 11686_2026_1391_MOESM1_ESM.docx]

**Integrated Experimental and Computational Investigation of Azobenzenoid Derivatives Against Trypanosomatid: RNA Editing Ligase 1 as a Potential Molecular Target**

Asif Khan^1^, Rodolfo Bento Balbinot^2^, Danielle Lazarin-Bidóia^2^, Maira Gabriela Paetzold^1^, Íris Nunes Seixas^3^, Gustavo Nunes Ramos Silva^4^, Maria Aparecida Fernandez^5^, Celso Vataru Nakamura^2^, Flavio Augusto Vicente Seixas^1*^

1. Department of Technology, State University of Maringá, Umuarama-PR, Brazil. (A.K.; M.G.P.; F.A.V.S.)
2. Technological Innovation Laboratory in the Pharmaceuticals and Cosmetics Development, State University of Maringá, Maringá-PR, Brazil. (R.B.B.; D.L.B; C.V.N.)
3. Department of Odontology, Paranaense University, Umuarama-PR, Brazil. (I.N.S.)
4. Department of Medicine, University of São Paulo, São Paulo-SP, Brazil. (G.N.R.S.)
5. Department of Biotechnology, Genetics and Cell Biology, State University of Maringá, Maringá-PR, Brazil. (M.A.F.)

*** Corresponding author:**

favseixas@uem.br

Universidade Estadual de Maringá - UEM

Laboratory of Structural Biochemistry

Department of Technology

Av. Ângelo Moreira da Fonseca, 1800, Pq Danielle

87506-370, Umuarama, PR, Brazil.

**Table S1**: Docking attempts to investigate CaCS2 (CID79465) pose frequencies across REL1 at different binding cavities.

| **Protozoa**  **REL1** | **Cavity A (orthosteric site)** | **Cavity B (n)** | **Cavity C (n)** | **Cavity D (n)** | **Cavity E (alternative site)** |
| --- | --- | --- | --- | --- | --- |
|  |  |  |  |  |  |
| *Leishmania* | 27 (random) | 0 | 0 | 0 | 23 (drug-like) |
| *Trypanosoma* | 50 (random) | 0 | 0 | 0 | 0 |

**Table S2.** Blind and guided docking attempts showing CaCS2 frequency across REL1 different binding cavities using different software packages.

| **Guided docking using different programs** | | | | |
| --- | --- | --- | --- | --- |
| **Pathogens** | **Target sites** | **Vina** | **AutoDock** | **Molegro** |
| *Leishmania* | CaCS2-alternative | -7.7 ± 0.00 | -9.9 ± 0.16 | -123.5 ± 0.32 |
|  | CaCS2-orthosteric | -9.0 ± 0.00 | -9.4 ± 0.37 | -128.2 ± 25.53 |
|  | ATP-orthosteric | -8.4 ± 0.00 | -7.3 ± 0.31 | -182.6 ± 21.28 |
| *Trypanosoma* | CaCS2-alternative | -7.7 ± 0.00 | -7.1 ± 0.06 | -95.4 ± 11.80 |
|  | CaCS2-orthosteric | -9.0 ± 0.00 | -10.3 ± 0.09 | -111.5 ± 3.10 |
|  | ATP-orthosteric | -8.4 ± 0.00 | -8.3 ± 0.90 | -173.0 ± 13.50 |

**Table S3**: The contact frequencies noted between *Leishmania-*REL1 and the ligands evaluated in this work by MD.

| **Residues** | **CaCS2-alternative site** | **CaCS2-orthosteric site** | **ATP-orthosteric site** |
| --- | --- | --- | --- |
| **Tyr-13** | - | 0.68 | **0.92** |
| Thr-14 | - | 0.16 | 0.37 |
| **Glu-15** | - | **0.87** | 0.37 |
| **Ile-16** | - | **1.00** | **0.84** |
| **Cys-40** | - | **0.90** | 0.09 |
| **Glu-41** | - | **0.89** | **1.00** |
| **Lys-42** | - | **1.00** | **1.00** |
| **Val-43** | - | **1.00** | **0.90** |
| His-44 | - | - | 0.01 |
| Gly-45 | - | 0.04 | 0.48 |
| **Thr-46** | - | **0.81** | 0.41 |
| **Asn-47** | - | **0.89** | **0.99** |
| **Arg-63** | - | **0.97** | **0.98** |
| Gly-111 | - | - | 0.09 |
| **Glu-112** | - | 0.52 | **1.00** |
| **Phe-160** | - | 0.75 | **0.81** |
| **Phe-162** | - | **1.00** | **1.00** |
| Ala-196 | - | 0.66 | 0.08 |
| Ile-216 | 0.11 | - | - |
| Pro-218 | 0.39 | - | - |
| Ala-221 | 0.74 | - | - |
| **Gly-226** | **0.99** | - | - |
| **Asn-227** | **0.95** | - | - |
| Pro-229 | 0.48 | - | - |
| Glu-236 | - | 0.04 | 0.24 |
| **Val-239** | - | **0.93** | **0.87** |
| **Arg-241** | - | **0.89** | 0.01 |
| **Lys-260** | - | **0.98** | **0.98** |
| **Arg-262** | - | 0.04 | **0.80** |
| Thr-304 | 0.18 | - | - |
| Asp-305 | 0.31 | - | - |
| Ala-306 | 0.50 | - | - |
| Met-307 | 0.47 | - | - |
| **Leu-308** | **0.85** | - | - |
| Pro-309 | 0.28 | - | - |
| Leu-311 | 0.05 | - | - |
| **Asn-372** | **0.96** | - | - |
| **Thr-373** | **0.81** | - | - |
| **Ser-374** | **0.87** | - | - |
| Leu-375 | 0.58 | - | - |
| Ser-376 | 0.05 | - | - |
| **Arg-378** | **0.81** | - | - |

Note: residues in bold have contact frequency greater/equal to 80%, while ‘-’ shows no contact. Only contact frequencies greater than 0.05 are shown.

**Table S4**: The contact frequencies noted between *Trypanosoma-*REL1 and the ligands evaluated in this work by MD.

| **Residues** | **CaCS2-alternative site** | **CaCS2-orthosteric site** | **ATP-orthosteric site** |
| --- | --- | --- | --- |
| **Tyr-52** | - | **0.99** | **0.99** |
| Ile-53 | - | 0.49 | - |
| **Glu-54** | - | **0.90** | 0.01 |
| **Ile-55** | - | **1.00** | **0.80** |
| Cys-79 | - | 0.01 | 0.27 |
| Glu-80 | - | 0.58 | 0.11 |
| **Lys-81** | - | **1.00** | **1.00** |
| Val-82 | - | 0.27 | - |
| His-83 | - | 0.22 | - |
| Gly-84 | - | 0.70 | 0.01 |
| **Asn-86** | - | 0.65 | **0.92** |
| **Arg-105** | - | **0.93** | **1.00** |
| **Glu-153** | - | **1.00** | **1.00** |
| **Gln-187** | - | **0.93** | - |
| Phe-201 | - | 0.12 | 0.23 |
| **Phe-203** | - | **1.00** | **1.00** |
| Asp-204 | - | 0.52 | 0.04 |
| Ser-237 | - | 0.04 | 0.78 |
| Lys-238 | 0.19 | - | - |
| Ala-239 | 0.03 | - | - |
| Leu-240 | 0.25 | - | - |
| Asn-255 | 0.69 | - | - |
| **Phe-256** | **0.89** | - | - |
| **His-257** | **1.00** | - | - |
| Thr-258 | 0.39 | - | - |
| **Pro-259** | **0.96** | - | - |
| Ala-262 | 0.33 | - | - |
| Leu-271 | 0.28 | - | - |
| Glu-272 | 0.15 | - | - |
| Gly-273 | 0.25 | - | - |
| Asn-274 | 0.25 | - | - |
| Glu-277 | - | - | 0.58 |
| **Val-280** | - | **0.96** | **0.98** |
| **Arg-282** | - | **0.98** | **1.00** |
| Arg-286 | - | 0.10 | 0.01 |
| **Ile-299** | - | **0.96** | **0.87** |
| **Lys-301** | - | 0.70 | **1.00** |
| Ile-342 | 0.43 | - | - |
| **Ala-343** | **0.85** | - | - |
| **Asp-344** | **0.84** | - | - |
| Ser-345 | 0.33 | - | - |
| **Met-346** | **0.92** | - | - |
| Leu-347 | 0.11 | - | - |
| Pro-348 | 0.06 | - | - |
| Gln-349 | 0.13 | - | - |
| Leu-408 | 0.61 | - | - |
| Ser-413 | 0.20 | - | - |
| Ala-415 | 0.01 | - | - |
| Phe-416 | 0.68 | - | - |

Note: residues in bold have contact frequency greater/equal to 80%, while ‘-’ shows no contact. Only contact frequencies greater than 0.05 are shown.
